# Homer 1 genotype AA variant relates to congenital splay leg syndrome in piglets by repressing Pax7 in myogenic progenitors

**DOI:** 10.3389/fvets.2023.1028879

**Published:** 2023-11-30

**Authors:** Toni Schumacher, Henry Reyer, Steffen Maak, Monika Röntgen

**Affiliations:** ^1^Institute of Muscle Biology and Growth, Research Institute for Farm Animal Biology (FBN), Dummerstorf, Germany; ^2^Institute of Genome Biology, Research Institute for Farm Animal Biology (FBN), Dummerstorf, Germany

**Keywords:** porcine congenital splay leg, PCS, SNP, homer1, myogenesis

## Abstract

**Introduction:**

Porcine congenital splay leg syndrome (PCS) is a major birth defect in piglets, resulting in lameness and high mortality rates. The multifactorial pathogenesis of PSC is not well understood but includes a polygenic inheritance.

**Methods:**

Here, in addition to morphological investigations, we characterized the expression of myogenic genes and functional (proliferation and differentiation) properties of myogenic precursor/satellite cells (SATCs) in 1 day-old PCS piglets, non-affected littermates (LCs), and piglets from PCS-free healthy litters (HCs). In addition, PCS phenotypes were related to the SNP Homer1_rs325197091 within the Homer1 locus, which has been identified as a potential hereditary cause of PCS.

**Results and discussion:**

Samples from *musculus semitendinosus* (ST) of PCS piglets had a higher proportion of type II fibers, reflecting myofiber immaturity. In addition, myofiber atrophy, a lower number of myonuclei per fiber (ST), and a higher apoptotic activity (in ST and longissimus dorsi muscle; LD) were found in the PCS group. A higher proportion of cycling committed myoblasts (Pax7^+^/Ki67^+^ cells) occurred in samples from PCS-affected piglets, and on the other hand, the mRNA expression of genes involved in differentiation (muscle differentiation 1; MyoD, myogenin; MyoG) was repressed compared with HCs. Cultured SATCs from PCS-affected animals showed a temporal shift in peak expression of Pax7, MyoD, and MyoG toward days 3 and 4 of their 7 days differentiation regime. *In vitro* experiments with isolated SATCs confirmed the lower differentiation potential and the delayed progression of the myogenic processes in cells from piglets with PCS phenotype. In addition, Pax7 and desmin were differently expressed in Homer1_rs325197091 genotype variants (GG, GA, and AA). Both genes showed the lowest expression in the homozygous AA-variant, which was most frequently found in PCS-affected animals. The homozygous AA-variant was also associated with lower expression of the truncated Homer1-subtype 205. Thus, we hypothesize that in PCS, the balance between Homer1 proteins and its signaling functions is changed in a way detrimental to the myogenic differentiation program. Our results demonstrated direct negative effects of the Homer1 AA genotype on Pax7 expression, but the exact mode of action still needs to be elucidated.

## 1 Introduction

Porcine congenital splay leg syndrome (PCS) is a transient locomotor disorder of newborn piglets resulting in muscle weakness with characteristic extension and lateral abduction of the hind and, more rarely, the front legs. The resulting immobility increases the risk of affected piglets being injured or dying from accidental crushing by the sow or from starvation (1). Thus, high incidence (0.4% to >8%) and mortality rates up to 50% make PCS a problem for the pig industry; first as a source of significant economic losses, but also due to the considerable negative impact on animal welfare and health (2).

PCS has long been recognized as a multifactorial disease (1, 3). Its pathogenesis is far from clear but involves a polygenic inheritance (4) as well as a multitude of environmental factors and stressors [reviewed in (2, 5)].

At the morphological level, no changes in the average size and shape of myofibers were found in neonatal PCS-affected piglets (1, 6, 7). Instead, the distribution pattern in the myofiber’s cross-sectional area (MCSA) shifted to smaller sizes in PSC muscles (8). However, myofibrillar hypoplasia (MFH), defined by a deficiency and irregular morphology of myofibrillar material in muscles of the trunk and limbs, has been frequently described in connection with PCS (5, 6, 9). Although more myofibers are affected by MFH in PSC piglets with clinical symptoms, degenerative processes have been rarely described, and some degree of MFH has also been found in healthy neonatal piglets (1, 10, 11). Altogether, these data are mostly interpreted as reflecting a reversible myofiber immaturity, mainly of secondary fetal fibers (2).

In contrast to the adult, neonatal, immature muscles are in an anabolic state, and protein synthesis is highly sensitive to acute changes in nutrient availability (12). During this period, a higher rate of myofibrillar than of sarcoplasmic protein synthesis is responsible for the rapid accumulation of myofibrils (12). Reduced availability of nutrients decreased the fractional protein synthesis; specifically, the accretion of myofibrillar proteins by 25%, respectively (12, 13). Indeed, Ooi et al. (8) found a downregulation of several structural MHC genes in 2 days-old PCS piglets. Moreover, prematurity has been shown to attenuate feeding-induced mTORC1 activation, translation initiation, and protein synthesis in the skeletal muscle of neonatal pigs (14, 15). Thus, low feed intake in PCS piglets will further impair myofibrillar development and decrease their chance of survival.

Conversion of secondary type II to type I fibers, and a characteristic grouped arrangement of the latter, is an important component of pre- and postnatal maturation of pig skeletal muscles (16, 17). Newborn piglets suffering from PCS had fewer type I fibers in their muscles than healthy controls (1). It has been shown that primary type I myofibers remain unimpaired by MFH (18), whereas secondary type II fibers are mostly affected (7). As myofiber-type differentiation is neurally regulated, altered motor innervation, defective excitation-contraction-coupling, or disturbed contractile mechanisms might cause myofiber immaturity (2, 11, 16).

In the pig, the number of myofibers is largely fixed at birth (19). Therefore, early postnatal muscle fiber growth critically depends on the sustained expansion and myonuclei accretion of so-called satellite cells (SATCs). SATC functionality is mainly regulated by various muscle-specific genes. Typically, SATCs express the marker-paired homeobox protein 7 (Pax7) and/or the determination factor myogenic factor 5 (Myf5) (20). In the adult muscle, only 1%–4% of the myonuclei belong to SATCs, and most of them are in a quiescent state. In contrast, the number of SATCs are much higher during the early postnatal period. In pig muscle, approximately 60% of the myonuclei are SATCs during the first week after birth, and of them, a high percentage (90%) is proliferating (21, 22). In the cycling myoblast population, Myf5 and/or MyoD are co-expressed with Pax7 (20). From these cells, myogenin (MyoG)-expressing fusion-competent myoblasts (approximately 30% of the population) are generated. SATC fusion contributes DNA to existing myofibers, thereby increasing their capacity for protein synthesis. We have shown that in neonatal piglets, a subpopulation of Pax7 expressing cells escapes from differentiation to form a pool of reserve cells that plays an important role in the establishment of the adult SATC population and for the replenishment of the proliferating population in fast-growing piglets (23). Pax7 and/or Myf5-positive SATCs are already found during prenatal development, and Pax7 precursor cells contribute to the growth of secondary muscle fibers *in utero* (19, 24). A subset of Pax7-expressing cells persist into the late fetal period, become enveloped by the basal lamina of developing myofibers, and establish the SATC pool (19, 25). Given the importance of these cells in prenatal and postnatal muscle development, it seems surprising that no investigations on the size, composition, and functionality of myogenic precursor populations in PCS have been carried out so far.

Evidence for the role of changed SATC functionality in PCS comes from investigations by Ooi et al. (8). These authors found myofiber atrophy characterized by upregulation of the E3 ubiquitin ligase MAFbx (FBXO3, atrogin) in longissimus dorsi (LD), semitendineus (ST), and gastrocnemius muscles of 2 days-old piglets with clinical signs of PCS. In contrast to MAFbx, P311, another muscle atrophy-associated gene, was consistently downregulated. P311 is typically expressed in myoblasts and stimulates proliferation, whereas terminal differentiation is delayed (8). Its expression increases during gestation and is high in normal growing muscle. Thus, reduced P311 expression in PCS muscles could lead to reduced availability of myoblasts for postnatal fiber hypertrophy. Hai et al. (26) confirmed muscle atrophy and strong upregulation of MAFbx at the transcriptional and protein levels in muscles of a pig mutant showing hind limb paralysis with a recessive inheritance pattern. Interestingly, MyoD and MyoG protein levels were strongly downregulated in mutants compared with wild-type piglets (26).

The ubiquitin-proteasome proteolysis pathway is implicated in the later stages of apoptosis (27), which is a normal process in pre- and postnatal muscle development. It has been shown that growth factor/serum deprivation, oxidative stress, DNA damage, and alterations in cell cycle progression or cellular metabolism can induce apoptosis in muscle cells (28). Replicative myoblasts are particularly affected, but their susceptibility to apoptosis depends on the cell cycle and differentiation stage as well as maturity level (29, 30). Induction of the cyclin-dependent kinase inhibitor p21, which is an important step in myogenesis (31), also results in a more apoptosis-resistant phenotype (32). In addition, IGF-II and BCL-2 are known survival factors and are expressed in neonatal and adult primary myoblasts (33, 34). In accordance with the role of apoptotic and protein degrading processes in PCS, Maak et al. (35) found several genes (SQSTM1, SSRP1, DDIT4, and MAF) involved in cell death to be differently expressed between PCS piglets and their healthy littermates.

A genome-wide association study (GWAS) identified four potential candidate genes for PCS, among them HOMER1 (4), which is highly expressed in muscle tissue (36). Further research on PCS-linked SNPs within the HOMER1 locus revealed a total of 19 SNPs, of which 12 were significantly associated with PCS. More importantly, 8 of these associated SNPs were located in intron 4, which functions as an alternative promotor for the truncated HOMER1 isoform 205. *In vitro* studies revealed a functional significance of Homer1_rs339135425, whose G-allele might create a new binding site for a transcription factor, as reported by promotor activity assays (37).

Common hypotheses on the pathogenic mechanisms involved in PCS phenotype development include a polygenic basis and muscular immaturity. However, myogenic progenitor cells/SATCs and their progeny, main components of muscle development, have never been investigated in PCS. Therefore, the main aim of this study is the characterization of molecular (myogenic genes) and functional (proliferation and differentiation) properties of the SATC population in PCS piglets compared to littermates without clinical phenotype and piglets from PCS-free litters. In contrast to other genes related to PCS, Homer1 has been shown to be involved in the control of various processes related to muscle development [reviewed in (5)]. Moreover, overrepresentation of certain alleles within the HOMER1 locus has been found in PCS-affected animals (37). To find out how differential expression of specific HOMER1 isoforms is involved in PCS pathogenesis, we aimed to explore possible effects on myogenic genes and, thus, the functionality of myogenic precursor cells.

## 2 Materials and methods

### 2.1 Animals

Animals were obtained from the experimental pig unit of the Institute of Farm Animal Biology. All experiments were conducted on material obtained from 1 day-old male piglets of the German Landrace that were slaughtered following the guidelines of the Animal Care Committee of the State Mecklenburg Western Pomerania, Germany, based on the Law of Animal Protection. Animals were euthanized by stunning the animals using a captive bolt before exsanguination. Since all manipulation and sampling on the animals were done after death, the experiments are not to be considered animal experiments. Sampling sessions were conducted every 3 weeks following the breeding cycles of the animal husbandry, and each included three animals, a PCS-affected piglet (PSC) that was selected according to a visible phenotype (lateral obduction of one or more limbs), a clinically non-affected littermate (LC), and a healthy control (HC) piglet from another litter free of PCS. Animals were also selected for matching birthweights (1.02 kg ± 0.15 kg). Overall, 20 sampling sessions were carried out, of which 63 animals were sampled for this study.

### 2.2 Histological methods

Directly after slaughter, tissue samples were excised from *musculus longissimus dorsi* (LD) and *musculus semitendinosus* (ST). Samples were then immediately snap-frozen in liquid nitrogen and stored until further use at −80°C. For histological analyses, serial transversal sections of 10 μm thickness were cut at −20°C using a cryotome Leica CM3050 S (Leica Microsystems, Wetzlar, Germany).

#### 2.2.1 Eosin staining for muscle fiber quantification

Freshly cut sections were directly mounted onto slides and allowed to dry at room temperature. The sections were then fixed for 5 min with a calcium-stabilized 4% formaldehyde solution. Fixative was rinsed off with distilled water. Cytoplasm was then stained for 5 min using 0.1% eosin solution. The excessive staining solution was rinsed off with distilled water for 5 min, and tissue sections were afterward dehydrated in an ascending alcohol series going from 70% (1 min) over 96% alcohol (1 min) to absolute alcohol (1 min) and a final rinse in Xylol (5 min). Sections were then covered in Eukitt^®^ (Merck, Darmstadt, Germany).

#### 2.2.2 Alkalic ATPase staining

To assess the muscle phenotype with regard to fiber type structure, alkalic ATPase staining was used to stain type II muscle fibers. By pre-incubation under either acid or alkalic conditions, the myosin-ATPase enzyme of specific fiber types gets inactivated, while during staining, the calcium atom of the remaining active enzyme is substituted by a cobalt atom, which will finally precipitate as a black insoluble compound. Freshly mounted cryosections were incubated at pH 4.2 for 8 min in acetic acid containing 180 mM CaCl_2_. The sections were then rinsed twice for 1 min with 100 mM Tris buffer containing 180 mM CaCl_2_ set to pH 7.8. Thereafter, a 60 min incubation with Na-ATP solution (180 mM CaCl_2_, 49.6 mM KCl, and 2.7 mM ATP disodium salt set to pH 7.8) at 37°C was performed. Then, the sections were incubated in a 2% CaCl_2_ solution for 3 min and rinsed four times for 30 s in distilled water. The final staining step was a 3 min incubation in 1% (NH_4_)_2_S solution for 3 min. The sections were again rinsed for 5 min under flowing tap water and finally rinsed in distilled water before getting mounted in glycerol jelly.

#### 2.2.3 Analysis of histochemically stained sections

The sections were photographed using an Olympus BX43 microscope with an Olympus UC30 camera attached (Evident Europe, Hamburg, Germany). Analyses were performed manually using a pen pointer and the Cell^D software suite (Evident, Hamburg, Germany). Ten fields of view of each specimen were analyzed (Olympus BCX43 with 20× objective; 0.48 mm^2^ FOV area) for muscle fiber quantification. ATPase cluster quantification was analyzed on 5 FOV per specimen (BCX43 with 10× objective; 1.47 mm^2^ FOV area).

#### 2.2.4 TUNEL assays

TUNEL assays were performed to quantify the amount of apoptotic activity in muscle samples using the “*In situ* Cell Death Detection Kit” (Merck, Darmstadt, Germany). Freshly cut and mounted cryosections were fixed with 4% paraformaldehyde/PBS for 20 min at RT. The fixative was washed off via PBS rinse for 30 min. The tissue was then treated with 0.1% Na-citrate solution. After a second washing step using PBS, the TUNEL mix was incubated for 60 min at 37°C on the sections. Finally, the sections were washed thrice with PBS and then mounted in Roti^®^-Mount FluourCare DAPI (Carl Roth, Karlsruhe, Germany).

#### 2.2.5 Immunofluorescence staining

Cryosections were mounted and fixed for 10 min using 4% paraformaldehyde PBS. The sections were then washed and permeabilized thrice for 10 min using PBS containing 0.1% Tween-20. After a further wash with PBS, the sections were blocked with 10% goat serum in PBS for 30 min. The sections were then incubated with primary antibodies against rabbit-anti-Ki67 (NB-600-1252: 1:90, Bio-Techne, Wiesbaden Nordenstadt, Germany) and mouse-anti-Pax7-c (DHSB; 1:45) overnight at 4°C. After additional washing with PBS, the sections were incubated with respective secondary antibodies (goat-anti-mouse-A-594 and goat-anti-rabbit-A-488; both 1:500 in 1% BSA-PBS) for 2 h at RT. After a final washing step with PBS (twice for 5 min), the sections were covered with Roti^®^-Fluor Care DAPI (Carl Roth). Microphotographs were taken using a Leica DM4000B epifluorescence microscope (Leica Microsystems, Wetzlar, Germany). Cell counting for DAPI-stained nuclei was performed using a semi-automated ImageJ script, while counting of Ki67 and Pax7 positive- or double-positive cells was conducted using ImageJ’s manual counting tool. For each sample, 10 randomly chosen FOVs were analyzed.

### 2.3 Myogenic progenitor cell isolation and cultivation

As described earlier (23), LD muscle was dissected and thoroughly digested with a Trypsin solution (4,000 U/mL) filtered and then administered to a Percoll gradient, consisting of 70%, 50%, 40%, and 25% layers. Two different subpopulations were isolated at the 50/70% interphase (subpopulation slow) and the 40/50% interphase (subpopulation fast). Cells were then re-suspended and cultivated in a growth medium (αMEM with 20% FBS, 100 U/mL penicillin/streptomycin, 5 μg/mL gentamycin, and 2.5 μg/mL amphotericin). For myogenic differentiation assays, cells from the 40/50 subpopulation were seeded on Matrigel (plastic ware coated with Matrigel diluted 1:50 and incubated for 1 h at room temperature) in differentiation medium (αMEM with 2% FBS, 100 U/mL penicillin/streptomycin, 5 μg/mL gentamycin and 2.5 μg/mL amphotericin) and were cultivated for 7 days. At each sampling day (0, 1, 2, 3, 4, 7), the cells were photographed and scraped off in TRIzol^®^ reagent (Thermo Fisher Scientific, Schwerte, Germany) for further gene expression studies.

### 2.4 Gene expression studies

#### 2.4.1 Sampling of RNA from muscle tissue

Muscle tissue from the ST was snap-frozen in liquid nitrogen directly after sampling and stored at −80°C until further use. Tissue samples of approximately 100 mg were put into a Xiril Dispomix tube together with 1 mL of QIAzol^®^ lysis reagent (Qiagen, Hilden, Germany) and were homogenized at 4000 rpm for 15 s. The homogenate was then centrifuged at 12,000 *g* for 10 min at 4°C. The supernatant was then mixed with 200 μL chloroform and mixed thoroughly for 15 s before being incubated for 3 min at room temperature. The mix was then centrifuged again at 12,000 *g* for 15 min at 4°C. Only the upper aqueous phase was afterward further processed for RNA precipitation by adding it to 500 μL isopropanol in a new micro-centrifuge tube and cautiously shaking the mix. RNA was then centrifuged at 20,000 *g* for 30 min at 4°C. The RNA pellet was once washed with 70% ethanol centrifuged at 7,500 *g* for 5 min at 4°C and then dried and eluted in 100 μL RNase-free water. The RNA was purified using a column-based RNA clean-up kit (Macherey-Nagel, Düren, Germany) according to the manufacturer’s instructions.

#### 2.4.2 Sampling of RNA from cell culture

Cells from satellite cell differentiation assays were washed with PBS once and layered with 500 μL TRIzol^®^ (Thermo Fisher Scientific, Schwerte, Germany) before being scraped off using a cell scraper. Cell samples were then stored at −80°C until further use. RNA was then isolated by standard guanidinium thiocyanate-phenol-chloroform extraction (38). In brief, 100 μL of chloroform was added to each of the TRIzol^®^ samples and vigorously vortexed. Samples were incubated for 5 min at room temperature and then centrifuged at 14,000 *g* for 15 min at 4°C. Afterward, the aqueous phase was mixed with 250 μL isopropanol in a new micro-centrifuge tube and briefly vortexed. The sample was incubated for 10 min at room temperature and afterward centrifuged at 20,000 *g* for 30 min at 4°C. The pellet was then washed with 500 μL of 70% ethanol and centrifuged at 7,500 *g* for 5 min at 4°C. The pellet was dried and then dissolved in 30 μL RNAse-free water. RNA content was measured using a photospectrometer (NanoDrop, Thermo Fisher Scientific), and the samples were stored at −80°C until further use.

#### 2.4.3 qPCR on samples derived from muscle samples and *in vitro* samples

qPCR primer design was facilitated with NCBI’s BLAST software suite. All primer pairs were designed to be exon junctions spanning in order to not anneal to potential genomic DNA contamination and were selected to have matching annealing temperatures to allow for multiple targets being processed with the same PCR protocol.

qPCR was performed using a qPCR kit (Vazyme, Düsseldorf, Germany) according to the manufacturer’s instructions on 12.5 ng of cDNA template using a standard cycle program with subsequent melting curve analysis on a Light cycler^®^ 96 (Roche, Mannheim, Germany). The fluorescence raw data from each run were analyzed using LinRegPCR software (v2018.0) for the calculation of proper qPCR efficiency values (39, 40). These were used to account for the amplification efficiency of the primer pairs (Table 1) using the equation given by Pfaffl (41) and expand it to incorporate expression data from multiple reference genes as their geometric mean (42). PPIA, CSNK2A2, and AP3D1 were used as reference genes in these analyses. The gene expression data were analyzed for fold change in PCS and LC piglets with respect to HC piglets.

**Table 1:** Primers for qPCR studies.

Gene	Reference sequence	Forward primer	Reverse primer
PPIA	NM_214353.1	CGCGTCTCCTTCGAGCTGTTT	GAAGTCACCACCCTGGCACAT
Csnk2A2	XM_021093947.1	AGTCTCACGTCCCGAGCTG	TGTTCCACCACGAAGGTTCTCC
Ap3d1	XM_021084198.1	CCTCATCCACAGCACGTCCG	GGCCAGGAGCCCCAGATACT
Desmin	NM_001001535.1	TTTGCTAGTGAGGCCAGCGG	GGATAGGGAGGTTGATCCGGC
Myf5	NM_001278775.1	TGTACCAAATGTATATGCCACGGAT	ATCGGTGCTGGCAACTGGAG
Pax7	XM_021095460.1	GACCCCTGCCCAACCACATC	ACATCCGGAGTCGCCACCT
MyoG	NM_001012406.1	CGCAGCGCCATCCAGTACAT	GCAGATGATCCCCTGGGTTGG
MyoD	NM_001002824.1	ACTGTTCCGACGGCATGATGGA	GCTCGACACCGCAGCATTCTT
Homer1-201	ENSSSCG00000014113	GGCAAAATGGGGGAACAACC	TTTGGGGTGATGGTGCTGTT
Homer1-205	ENSSSCG00000014113	CAAAAGAATCAGCAGGCGGG	CCAGTTCAGCCTCCCAATGT

#### 2.4.4 Single nucleotide polymorphism detection and genotyping

Liver tissue was used to isolate genomic DNA. Samples were lysed with proteinase K (Carl Roth). A phenol-chloroform extraction was performed using Phase Lock Gel tubes according to the manufacturer’s recommendations (5 Prime, Hamburg, Germany). The genomic DNA was precipitated, re-suspended in Tris-EDTA buffer, and stored at −20°C until further use. According to previously published results (37), an SNP in the intronic region of HOMER1 was selected (rs325197091). Polymerase chain reactions (PCRs) were performed in a 20 μL volume containing 100 ng of genomic DNA with SupraThermTaq DNA polymerase (GeneCraft, Münster, Germany). TCAGCCAATCAATAAATAACCTTG and TATCCTATACAC GTTTCTTGTGCTG were used as primers, resulting in a PCR product with a fragment length of 809 bases. The touchdown PCR had the following specifications: initial activation at 95°C for 5 min followed by 10 cycles of 15 s at 95°C for denaturation and 60 s at 63°C–58°C (drop of 0.5°C per cycle) for annealing and elongation. Subsequently, 30 cycles of 15 s at 95°C, 1 min at 58°C, and 1 min at 72°C were performed, followed by 5 min at 72°C. The PCR products were assessed for specific amplification on 1.5% agarose gels. Products were digested by the restriction enzyme Xcel (Thermo Fisher). Corresponding reaction conditions were 16 h at 37°C for incubation and 20 min at 65°C for enzyme inactivation. The retrieved fragments were separated into 3% agarose gels and evaluated.

### 2.5 Statistical analyses

Statistical analyses on the respective readouts were conducted using the GraphPad Prism 8 software suite (GraphPad Software, Boston, United States). Bar graphs show means plus standard deviation. Statistical difference between groups was computed via one-way ANOVA with Tukey’s multiple comparison test. A two-sided Fisher’s exact test was used to compare the allelic distribution at the HOMER1 locus between control and PCS-affected animals. Group differences were considered to be significant at *p* ≤ 0.05. Significance levels are marked as follows: “^*^” for *p* ≤ 0.05; “^**^” for *p* ≤ 0.01; and “^***^” for *p* ≤ 0.001.

## 3 Results

### 3.1 Muscle structure, myofiber typing, and TUNEL assay

**Figure 1:**
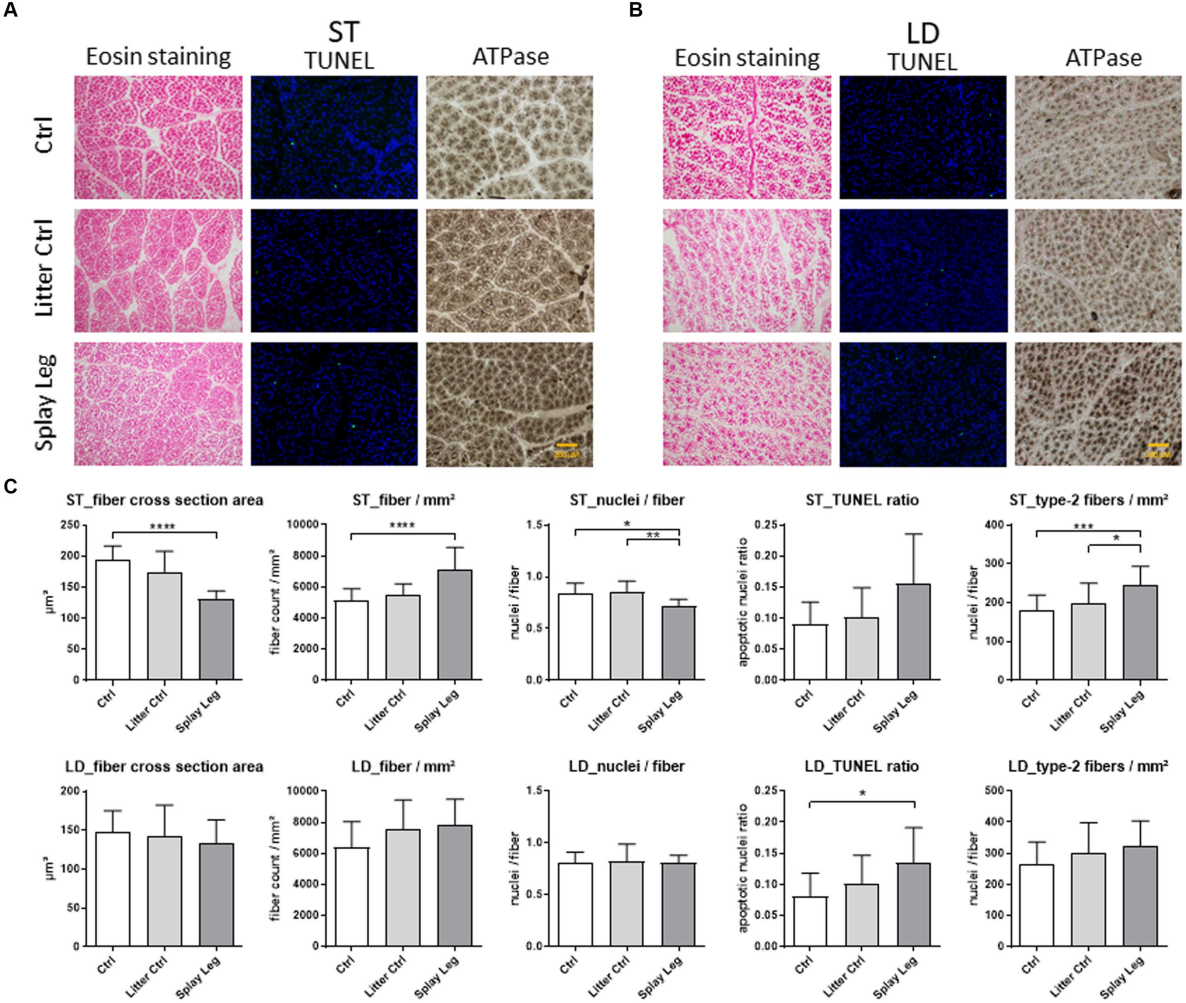
Representative slides stained with either eosin (left), TUNEL-assay (middle), or alkalic ATPase (right) from samples of 1 day-old control (upper row), litter control (middle row), or splay leg piglets (lower role) of *musculus semitendinosus* (ST, **A**) and *musculus longissimus dorsi* (LD, **B**). Diagrams **(C)** show quantitative analysis results for muscle structure, apoptotic activity (TUNEL ratio), and type II myofiber numbers for both muscles. Asterisks denote significant differences between the groups (^*^p < 0.05, ^**^p < 0.01, and ^***^p < 0.001).

Myofiber cross-sectional area (MCSA), myofiber density, myonuclei per fiber, and type II myofibers were determined for all groups. In addition, TUNEL assays were performed to see whether apoptotic processes might play a role in functional disorders observed in PCS piglets. Representative ST (Figure 1A) and LD (Figure 1B) histological sections and summarized results are shown in Figure 1C.

Significant structural changes were found in the ST of 1 day-old PCS piglets only (Figure 1C). Specifically, the MCSA was strongly reduced in PCS-affected animals compared to HC piglets. In accordance, significantly more myofibers were found per analyzed area in the PCS group. In addition, the number of nuclei per myofiber reflecting mostly SATCs was significantly lower in piglets with PCS phenotype than in LC and HC piglets.

ATPase staining revealed a higher number of type II muscle fibers in the ST of PCS-affected animals compared to LC and HC piglets (Figure 1C).

Although apoptotic activity was increased in ST and LD muscles of PCS piglets (Figure 1C), significant cell death was observed in the samples from the LD of PCS animals only.

### 3.2 PAX7 and Ki67 protein abundance in ST and LD muscle samples

**Figure 2:**
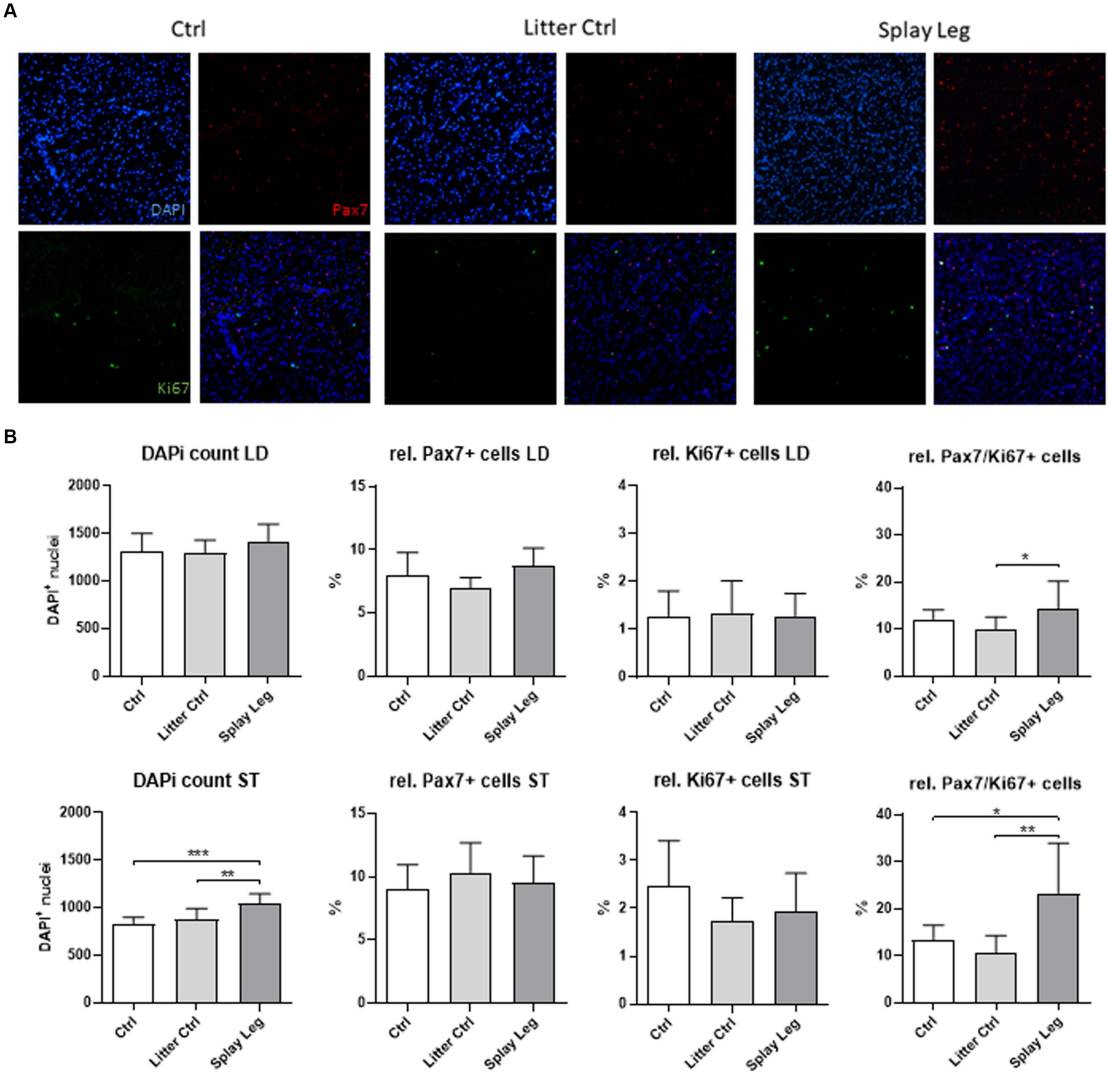
Representative slides of muscle samples stained for Pax7 (red), Ki67 (green), and DNA (blue) **(A)**. Quantitative analysis of samples from 1 day-old control, litter control, and splay leg piglets **(B)**. Asterisks denote significant differences between the groups (^*^p < 0.05, ^**^p < 0.01, and ^***^p < 0.001).

Because a lower number of nuclei per myofiber (ST) and a higher degree of cell death (LD) were found, we investigated the population of myogenic progenitor cells in more detail by the quantification of total nuclei and the proportion of Pax7^+^/Ki67^−^ SATC, Pax7^+^/Ki67^+^, and Pax7^−^/Ki67^+^ myoblasts (Figure 2A). The total myonuclear number was similar for all groups in LD samples, whereas it was increased in the PCS group compared with both control groups in samples from ST (Figure 2B). However, in both muscles, PCS animals showed significantly higher numbers of Pax7/Ki67-double positive myoblasts when compared to either LC (LD muscle) or even both control groups (ST muscle) (Figure 2B).

### 3.3 Myogenic gene expression in muscle samples

Next, we investigated a set of myogenic genes (Pax7, Myf5, MyoD, MyoG, and desmin) in samples from the ST muscle.

**Figure 3:**
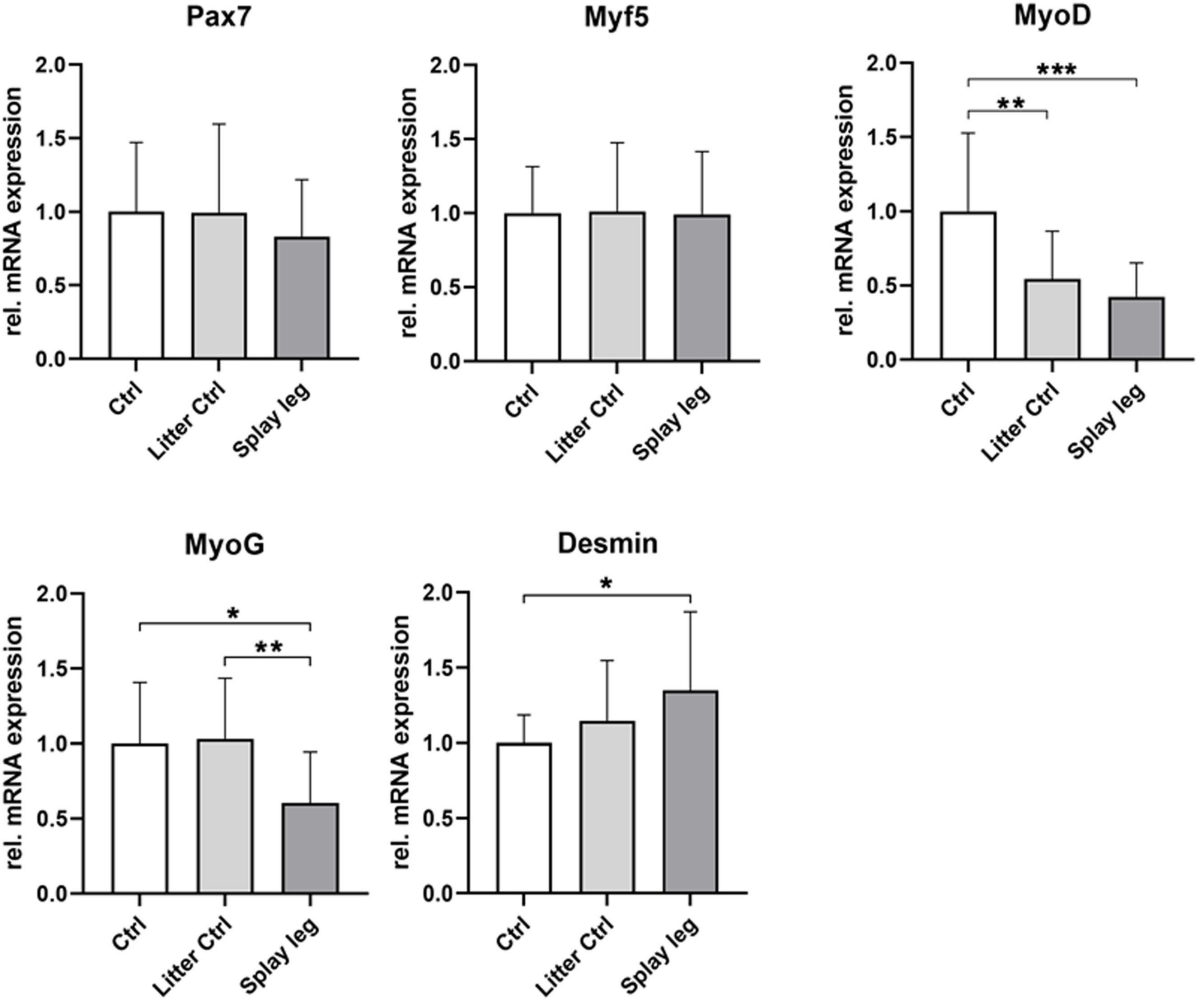
Relative mRNA expression of muscular differentiation markers Pax7, Myf5, MyoD, MyoG, and desmin in *musculus semitendinosus* of 1 day-old piglets. Asterisks denote significant differences between the groups (^*^p < 0.05, ^**^p < 0.01, and ^***^p < 0.001).

As shown in Figure 3, the expression level of Myf5 and Pax7 was similar in the PCS and control groups, whereas the expression of the proliferation marker MyoD was significantly reduced in the PCS and LC groups. In addition, MyoG, which is expressed during early differentiation, showed a significant decline in mRNA expression in PCS compared to LC and HC animals (Figure 3). On the other hand, increased expression levels of desmin were found in PCS animals compared with HC piglets.

Lower RNA expression levels of MyoD (HC and PCS) and MyoG (PCS only) point to a repressed differentiation potential of myogenic precursor cells from piglets originating from litters with PCS.

### 3.4 *In vitro* studies on differentiation capacity of isolated muscular satellite cells

**Figure 4:**
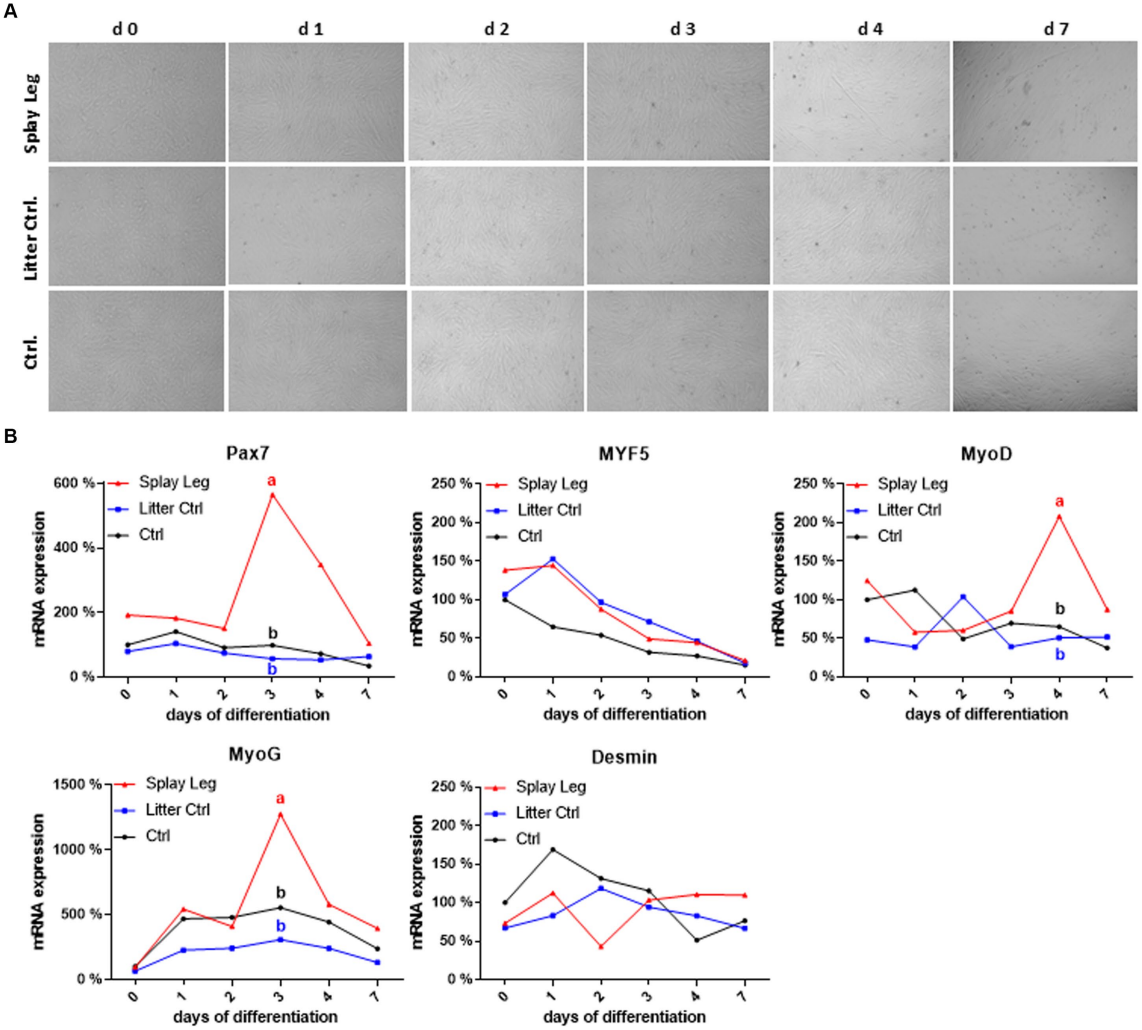
Light microscopy of differentiating porcine primary satellite cells from *musculus longissimus dorsi* during myogenic differentiation over 7 days **(A)**. mRNA expression profiles of selected myogenic markers during differentiation **(B)**. Different lowercase letters denote significant differences between the groups at *p* < 0.05.

To determine whether the observed negative effects on myogenic genes involved in muscle differentiation are a result of unfavorable systemic conditions or actual cell-intrinsic physiological deficits, we chose an *in vitro* approach to explore growth and differentiation in primary isolated muscle progenitor cells from LD muscle. While overall cell proliferation capacity was unchanged in all groups (data not shown), isolated cells from PCS-affected piglets showed a delayed differentiation process. Therefore, we cultured cells from all groups under conditions promoting differentiation (growth factor reduction) and investigated temporal changes in the transcription profile of the abovementioned myogenic genes at day 0 (initial day of differentiation assay), 1, 2, 3, 4, 5, and 7 after induction. Regardless of their origin, cells were able to differentiate and fuse, as shown by myotube formation (Figure 4A). However, qualitative microscopic analysis showed less pronounced myotube formation within the PCS group on days 4 to 7 compared to the HC group.

In both control groups, Pax 7 expression was relatively constant, whereas Myf5 expression decreased continuously in cells from HC only (Figure 4B). As expected, a reduction of MyoD expression (stabilizing at approximately 50% of the initial value) and an increase in myogenin expression reaching its maximum at day 3 characterized the differentiation process in HC. In contrast, Myf5 expression increased from day 0 to day 1 in LC, which also showed a delayed and shorter upregulation of MyoD, reaching a peak at day 2. The kinetics of MyoG expression was similar in both control groups, with somewhat lower expression levels in cells from LC piglets. In comparison to HC cells, those from PCS-affected animals had higher initial levels of Pax7 and Myf5, and as for LC controls, Myf5 downregulation started later (at day 1). In addition, the expression kinetics of Pax7, MyoD, and MyoG were characterized by marked peaks in PSC cells. For Pax7 and MyoD, those peaks occurred on days 3 and 4, respectively, when expression levels were low in both control groups. Although the kinetics of MyoG expression was similar for all groups, showing a peak expression at day 3, it was significantly higher in PSC compared to both control groups. In contrast, Myf5 and Desmin did not show significant differences between the groups (Figure 4B).

### 3.5 Studies on Homer1 genotypes, isoform expression, and effects on myogenic development

A recent study suggested a link between a specific SNP at the Homer1 locus (rs325197091) and the occurrence of PCS. This SNP was located in an intronic region, which was identified as a potential promotor for a truncated Homer1 isoform (37).

We, therefore, genotyped our test samples at this locus. The genotype frequencies in the control animals were 0.23 (GG), 0.48 (GA), and 0.29 (AA). The allele frequency was shifted from 0.51 (G) and 0.49 (A) in control animals to 0.39 (G) and 0.61 (A) in the PCS-affected animals. This difference, however, was not significant (*p* = 0.1993), which is likely due to the limited sample number.

**Figure 5:**
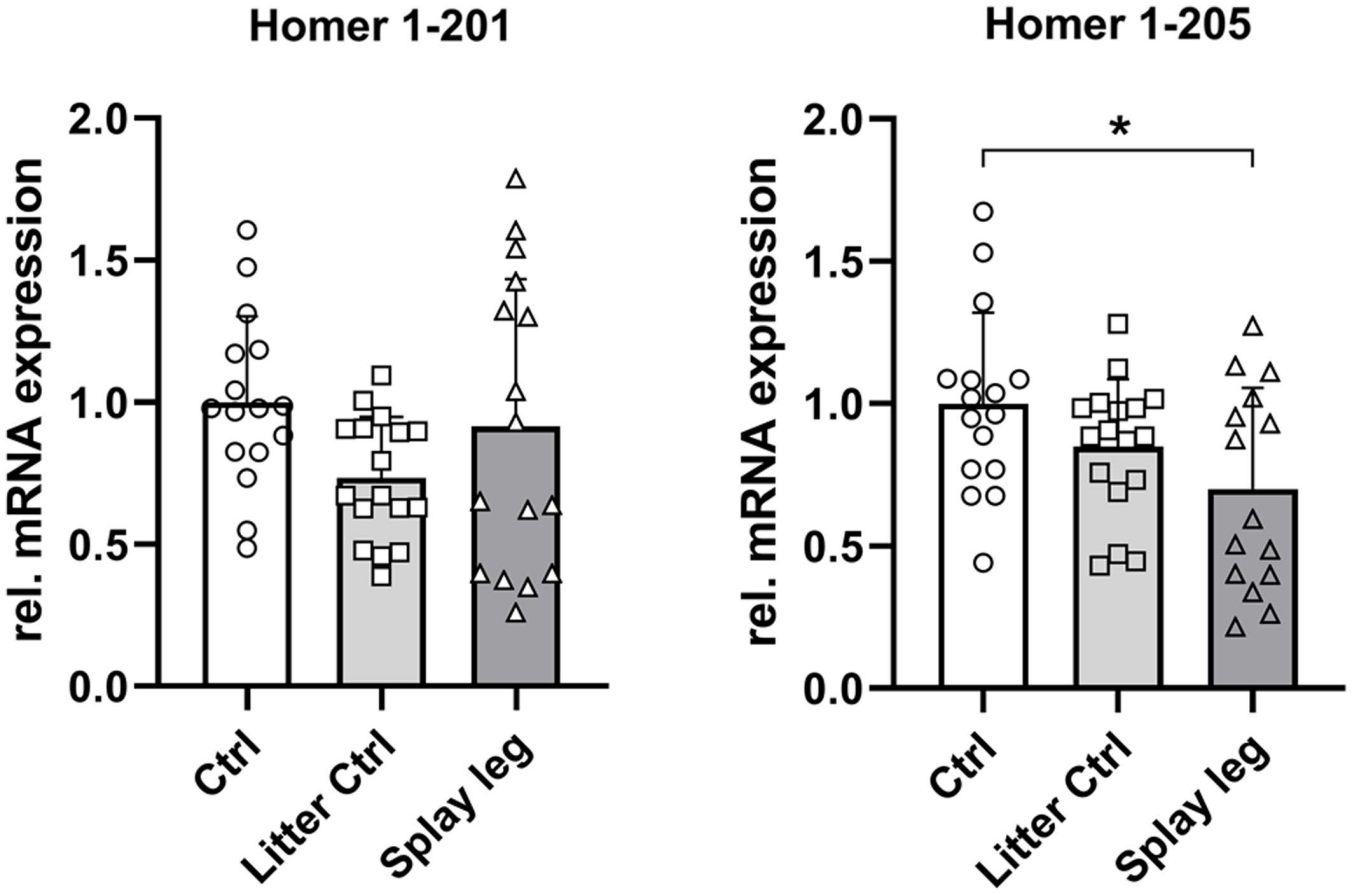
Relative mRNA abundance of Homer1 isoforms 201 and 205 in muscle samples of control, litter control, and splay leg piglets (^*^significantly different at *p* < 0.05).

Next, we analyzed the relative mRNA abundance of the full-length Homer1 isoform 201 and the truncated form 205. Quantitative PCR revealed no significant difference in the expression of the full-length isoform 201. In contrast, Homer1-205 expression was significantly lower in PCS-affected piglets than in HC piglets, whereas the difference between LC and PCS was not significant (Figure 5).

To test for the consequences of different Homer1 SNP genotypes on the expression of the Homer1 isoforms, we re-grouped all the samples accordingly.

**Figure 6:**
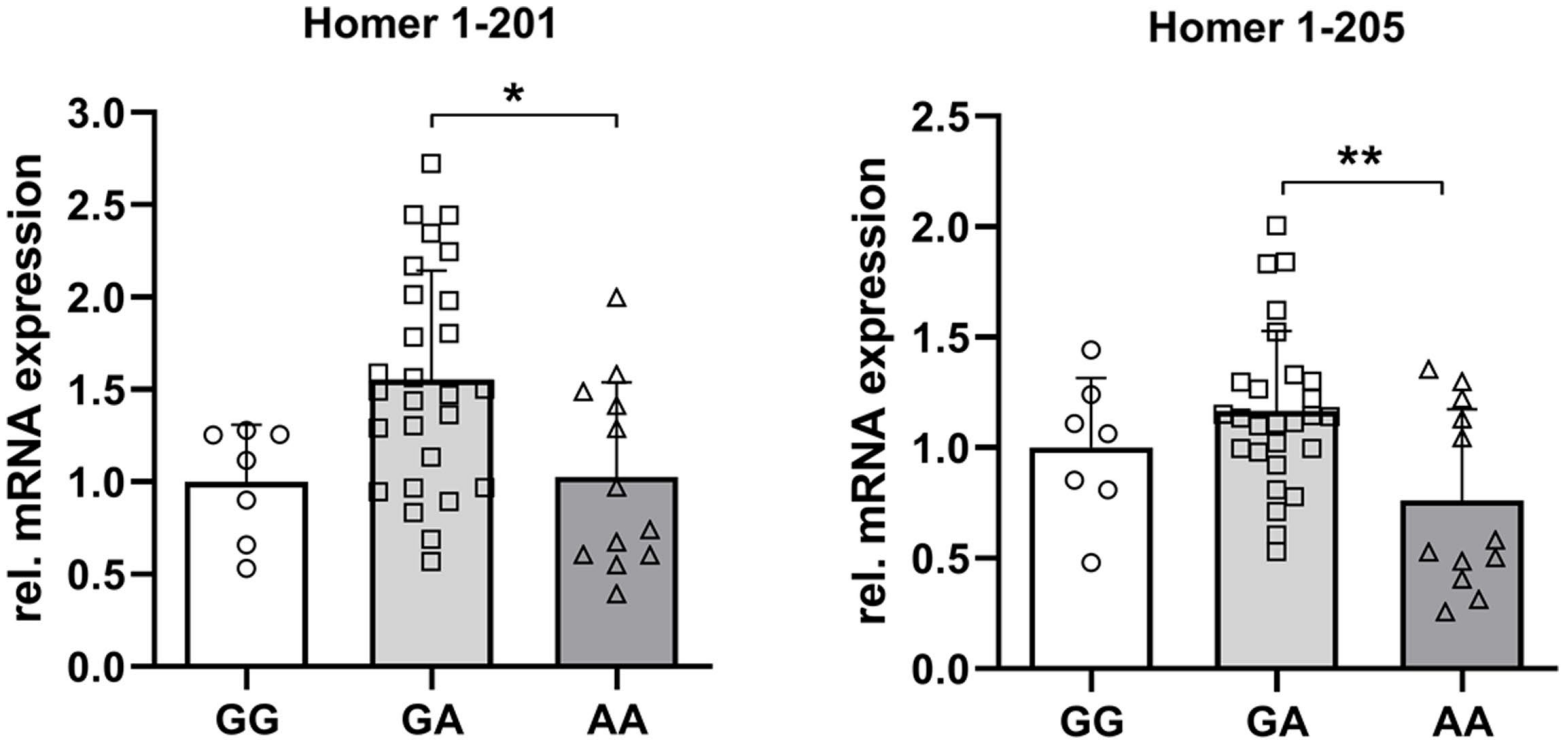
Relative mRNA abundance of Homer1 isoforms 201 and 205 dependent on the genotype at the SNP rs_325197091 of the porcine Homer1 locus. Asterisks denote significant differences between the groups (^*^p < 0.05 and ^**^p < 0.01).

The expression of the full-length transcript of Homer 1 (201) was almost identical in GG and AA genotypes. However, the expression of Homer1-205 was 24% (*p* = 0.18) and 41% (*p* < 0.01) lower in the AA compared to GG and GA genotypes. These results may indicate a functional relevance of this specific SNP on the expression of the truncated Homer1-isoform (Figure 6).

Next, we reassigned the expression levels of myogenic genes (data shown in Figure 3) to the Homer1 genotypes.

**Figure 7:**
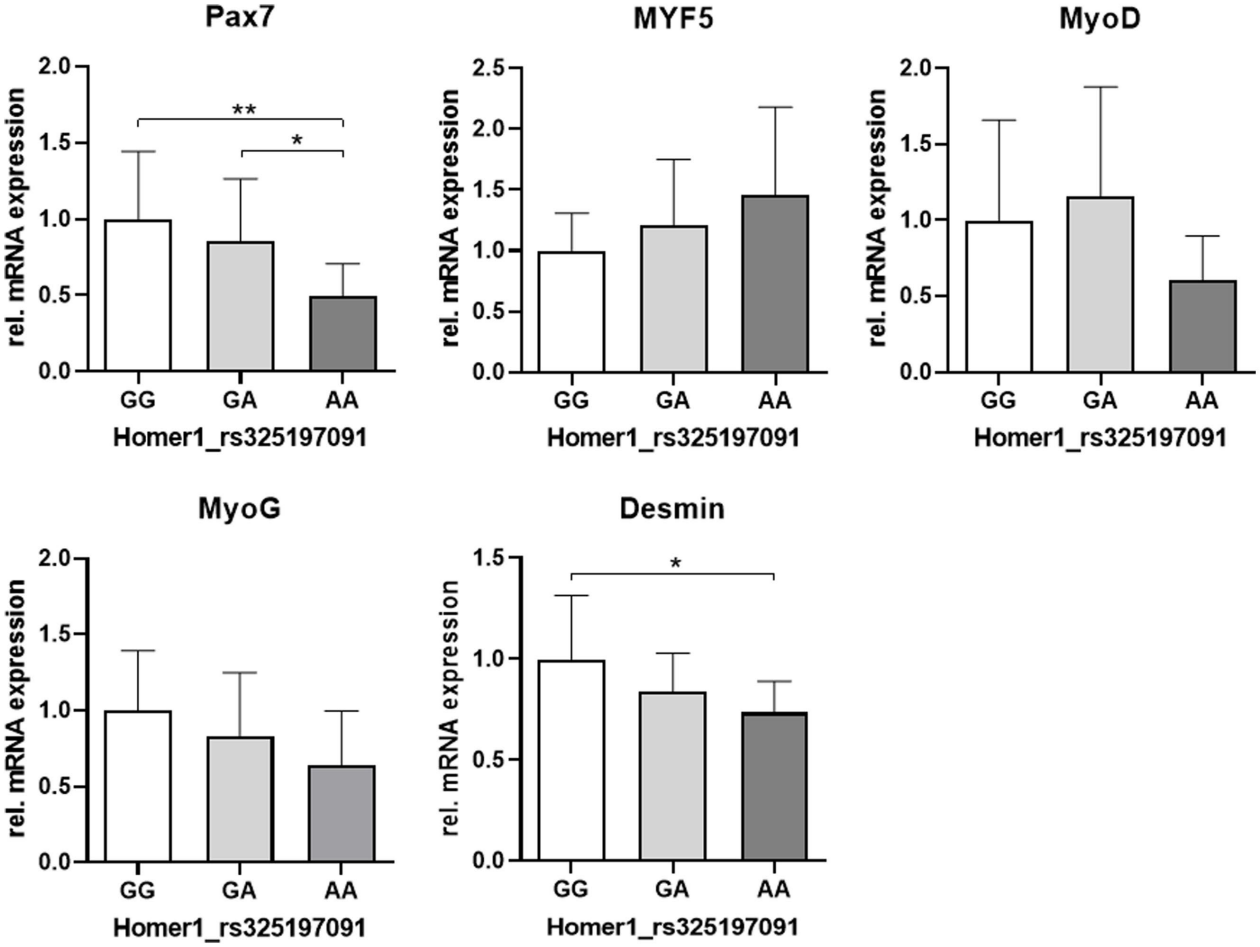
Relative mRNA abundance of myogenic markers related to the genotype at the HOMER1 locus. Asterisks denote significant differences between the groups (^*^p < 0.05 and ^**^p < 0.01).

The expression levels of the SATC marker Pax7 and desmin were related to the Homer1 genotype, with the lowest expression in the AA variant. For MyoG and MyoD expression, a similar trend was observed (Figure 7).

## 4 Discussion

In our study, we used muscle tissues (ST and LD) and isolated SATCs (LD) from 1 day-old piglets with clinical PCS and respective controls (LC and HC) to characterize muscle tissue parameters, as well as molecular and functional properties of the SATC population. In addition, the relationship between those properties with Homer1 genotypes was explored.

### 4.1 Reduced fast-to-slow myofiber switch, myofiber atrophy, and increased apoptotic activity characterize muscles from 1 day-old PCS piglets

LD and ST muscles were selected for our study as those muscles are mostly affected by PCS (7–9). However, the LD muscle is a light muscle containing mostly type II myofibers (17), while the ST is composed of a superficial light and a deep dark part (43). In addition, the kinetics of maturation processes and the growth rates differ markedly between LD and ST muscles (44).

For example, type II to type I transformation in the deep portion of the ST starts already during late fetal development, whereas it begins only after birth in LD (16, 45). Consequently, at birth, the ratio between type II and type I fibers amounts to 5.7 and 26.0 in normal ST and LD muscles, respectively (16, 17). Our data show a significantly higher number of type II myofibers in the ST of 1 day-old PCS piglets compared with both control groups. These results point to a reduced fast-to-slow fiber-type switch in the PCS group, reflecting a strong negative effect on ST prenatal myofiber maturation (11). MEK1-ERK1/2 and calcineurin (CN)/nuclear factor of activated T cells (NFAT) signaling pathways are involved in the activation of a slow, oxidative program in muscle progenitors and myofibers (46, 47) and could be disturbed in PCS piglets. Promoting type I muscle fibers, and thereby an oxidative, slow-twitch phenotype, has been shown to protect skeletal muscles from muscle weakness, and it reduced the progression of Duchenne muscle dystrophy in humans (46, 47). The higher resistance of type I myofibers is among other factors related to increased expression levels of utrophin A, which is also regulated in a CN/NFAT-dependent manner (48, 49). Thus, fewer type I myofibers in the ST muscle, which is used for locomotion, might contribute significantly to the prenatal/neonatal PCS phenotype. This could be explained by a generally increased risk for type II myofibers to be affected by MFH such that the critical threshold leading to muscle weakness is exceeded (7, 50). In addition, the myofiber metabolic type also affects the SATC number and reserve cell properties. Type II fibers have fewer SATCs that show a higher potential for self-renewal and start terminal differentiation later than those from type I fibers (51).

Like other authors (8, 26, 52), we found a smaller mean MCSA along with higher numbers of myofibers in ST samples from PCS affected compared with HC piglets. Thus, 1 day-old PSC piglets showed a marked myofiber atrophy in ST but not in LD muscle. This finding relates to the fact that ST and LD differ greatly in their pre- and postnatal growth rates (44), which *per se* determines the variable morphology of neonatal ST or LD muscles at birth. In addition to the intensive synthesis of myofibrillar proteins, the prenatal growth process includes a strong increase in the total number of nuclei. Most of them were provided as new myonuclei to growing secondary fibers, resulting under normal conditions in an 18% reduction of the SATC population until day 1 after birth (19). Muscles of the hind limbs show a much higher growth rate (approximately 3-fold) than LD muscle during prenatal development. Moreover, of all muscles, the prenatal growth rate is lowest in the LD, resulting in the lowest MCSA at birth (44). Thus, in accordance with our results, disturbed prenatal growth will have stronger effects on fast-growing muscles, such as the ST. Here, in addition to reduced myofiber growth, we found a lower number of myonuclei per fiber in ST muscle samples from 1 day-old PSC piglets compared with both control groups. This points to impaired functionality and/or loss of SATCs, specifically during the period of intensive growth of secondary myofibers before birth (19). Interestingly, in most studies with neonatal PCS piglets, MFH but not myofiber atrophy was found in a wide range of muscles (1, 6, 7). Moreover, in the LD, significant PCS-related myofiber atrophy was only found when investigated piglets were at least 2 days old (8, 26, 52). The LD is the only muscle with an increased growth rate after birth, and moreover, its fiber size increases the most (44, 53). Thus, the occurrence of negative effects on myofiber hypertrophy is related to the onset of postnatal SATC-dependent growth in LD.

Apoptotic processes are known to precede measurable myofiber atrophy (54). Indeed, by using the TUNEL assay, we found a significantly higher (LD) or numerically increased (ST) apoptotic activity in PCS-affected animals compared to HCs. Apoptosis is a rapid process, and affected cells are immediately phagocytosed (27, 54). Thus, even the apparently small number of apoptotic cells in our study indicate massive cell death in the PSC group. In contrast to necrosis, apoptosis is gene-dependent. In accord, several genes involved in cell death, specifically the atrophy marker FBXO32 (MAFbx), have been shown to be upregulated in muscle from piglets with clinical PCS symptoms (8, 26, 35, 52).

Myoblasts are most sensitive to apoptosis, specifically during transition into differentiation (29, 32, 55). Therefore, an elevation of the TUNEL-positive fraction of muscle cells might occur when the normal interplay between pathways that regulate proliferation and differentiation is disturbed. For example, increased MAFbx expression in PCS-affected muscles led to the downregulation of MyoD and MyoG protein levels and MyoD degradation, resulting in defective myoblast differentiation (26, 56).

Thus, in the next step, we investigated the cycling progenitor population in more detail.

### 4.2 A population of proliferating Pax7 positive progenitors dominates in the tissue of PCS piglets

In the pig, a high proportion of the SATC/progenitor population expresses Pax7 in the juvenile proliferative phase, but these cells can differ in their differentiation status. Thus, we stained the progenitor cells with both Pax7 and Ki67, a marker of actively dividing cells, and evaluated the proportion of Pax7^+^/Ki67^−^, Pax7^+^/Ki67^+^, and Pax7^−^/Ki67^+^ cells. The Ki67-negative population might represent non-activated Pax7^+^ SATCs, whereas Pax7^+^/Ki67^+^ and Pax7^−^/Ki67^+^ cells are activated (20, 57, 58).

The proportion of Pax7^+^/Ki67^−^ to total nuclei was similar in all groups and in both muscles. This population reflects cells in which Pax7 had induced withdrawal from differentiation and transition into quiescence (59, 60). In juveniles, this process is important for forming the adult SATC pool (59). In addition, SATCs with high Pax7 expression form so-called reserve cells that are needed to replenish the SATC pool but also give rise to fast-proliferating cells with higher differentiation potential (23, 61). Interestingly, the proportion of PAX7^+^/Ki67^−^ cells found in this study (approximately 10%) is in good agreement with *in vitro* data from Patruno et al. (62). The authors isolated SATCs from the ST of newborn piglets, and after 1 week in culture, they found 13.6% of cells positive for Pax7. Thus, in accordance with the reversible nature of the PCS syndrome (18), the relative proportion of reserve cells is maintained in the PCS group.

However, the proportion of Pax7^+^/Ki67^+^ cells was markedly higher in the PCS group of both muscles when compared to LC (LD) or both control groups (ST). Thus, PCS favors the generation of proliferating committed progenitors which together with Pax7 express the myogenic genes Myf5 or MyoD (58, 63). This seems to be a characteristic of muscles from piglets with clinical signs of the syndrome because the proportion of Pax7^+^/Ki67^+^ myoblasts is rather reduced in samples from the LC group in both muscles. This shift can be interpreted to reflect a reduced differentiation potential in the myogenic progenitor population of PCS piglets (60). However, the proportion of Pax7^−^/Ki67^+^ cells was similar in all groups for the LD and only numerically reduced in the LC and PCS groups of the ST.

Therefore, to get more direct information on the differentiation potential, we next investigated the mRNA expression of a wider range of myogenic genes in samples from ST muscle.

### 4.3 Lower expression of the myogenic master regulator MyoD at the mRNA level is a main characteristic of PCS piglets and litter controls

In tissue samples from ST, the mRNA expression of Pax7 and Myf5 was similar over all groups, whereas the expression level of MyoD was markedly reduced in samples from both LC and PCS piglets compared with HCs. A large number of differentiation-related genes, e.g., creatine kinase, acetylcholine receptor, MyoG, the cyclin-dependent kinase inhibitor p21, and myosin light chain, are MyoD targets and can be negatively affected by MyoD repression (31, 64–68). Thus, lower MyoD expression in LC and PCS groups means that all piglets from affected litters have an increased risk of developing a visible splay leg phenotype due to regulatory disorders at various levels of the differentiation process. Here, we found that the expression of MyoG was normal in LC but significantly reduced in PCS piglets. MyoG is crucial for generating fusion-competent myoblasts and, thus, hypertrophic myofiber growth. Therefore, MyoG downregulation might contribute to myofiber atrophy observed in the ST of PCS but not LC piglets.

In the muscles of newborns, desmin has been identified as a hallmark of replicating myoblasts (69, 70). In this study, we found desmin expression to be significantly upregulated in ST tissue from the PCS compared with the HC group. This is in accordance with our finding of a higher proportion of Pax7^+^/Ki67^+^ myoblasts in the muscle tissue of PCS piglets. Thus, different from the LC group, a majority of newly generated myoblasts escape terminal differentiation in the PCS group, which also makes them more sensitive to apoptosis (29). However, it remains to be clarified whether and how other MyoD targets besides MyoG also contribute to the expression of the clinical PCS phenotype.

Because a multitude of extrinsic factors such as nutrition can affect SATC functionality *in vivo*, we performed *in vitro* differentiation assays with isolated cells and evaluated the same panel of myogenic genes to elucidate whether cell-intrinsic SATC properties are affected in piglets with PCS syndrome.

### 4.4 *In vitro*, cells from PCS piglets generate cycling committed myoblasts showing delayed differentiation

It has to be mentioned that observed group differences in the mRNA expression levels at day 0 (induction of differentiation by serum reduction from 20% to 2%) of the experiment might indicate differences in the composition of the SATC population. Consistent with the results on Pax7 and Ki67 protein abundance in LD tissue samples, higher mRNA levels of Pax7 and MyoD seem to reflect a dominant population of proliferating Pax7^+^/MyoD^+^ cells in isolates from PCS piglets, whereas this population seems to be much smaller in cells from LC piglets.

As seen in the HC group, Myf5 expression normally declines when cells start to differentiate (71, 72). However, Myf5 expression is higher in PCS (day 0 to 1) and LC (day 1) piglets at the beginning of the experiment, and its downregulation during the experimental period is slower than in HC piglets, which will delay the entry into the differentiation process in both groups (72). Specifically, cells isolated from LC piglets upregulate Myf5 from day 0 to day 1, which could reflect reserve cell formation (73). This will also explain the small reduction of MyoG expression in the LC compared with the HC group, whereas the MyoG expression kinetics is similar. In a positive way, this could be interpreted as an adaptive response directed to regenerate the reserve cell/SATC pool by limiting terminal differentiation.

However, striking changes in the expression kinetics of Pax7, MyoD, and MyoG were found in the PCS group. At the beginning of the experiment, part of the cells from PSC piglets seemed to react normally (downregulation of MyoD and upregulation of MyoG) to the differentiation medium. However, at later time points, cells from PCS piglets showed extreme expression peaks of Pax7, MyoD, and MyoG, which were not observed in any control groups. Especially, upregulation of Pax7 and MyoG at the same time point was surprising because their expression is known to be mutually exclusive during differentiation (60). Therefore, the only reasonable explanation for this observation is a transient increase of committed myogenic progenitors (Pax7^+^/MyoD^+^) and cells in a very early phase of differentiation (MyoD^+^ and/or MyoG^+^), whose transition into terminal differentiation is delayed. In accord, microphotographs of these differentiating cultures also show less organized myotube formation at the end of the differentiation regimen.

### 4.5 Homer1 AA variant is linked to PCS pathogenesis by lowering Pax7 expression

Homer1, due to its role as a scaffolding and adaptor protein, is a crucial player in a multitude of intracellular signaling cascades involved in myogenic maturation. It is thus not surprising that Homer1 has been considered a candidate gene for PCS heredity in previous genetic studies on PCS (4). In this study, genotyping of muscle samples from PCS-affected animals revealed a trend toward a higher frequency of the A than the G alleles in the Homer1_rs325197091 SNP in contrast to the results of Xu et al. (37), who observed a significantly higher frequency of the G allele in a larger sample of splay leg piglets. However, the A-allele was significantly linked to a decreased expression of the truncated Homer1-205 isoform in our study, which is in line with the observed higher promoter activity in piglets bearing the GG genotype (37).

Surprisingly, the expression of the SATC marker Pax7 was significantly reduced in piglets with the genotype AA at the Homer1 locus, whereas a comparison of the phenotypic groups (HC, LC, and PCS) did not reveal any significant changes. Thus, our results indicate a direct link between the myogenic factor Pax7 and PCS pathogenesis. This is substantiated by Seale et al. (74), showing that mice with Pax7 null mutation (Pax7^−/−^) developed muscle weakness characterized by an abnormal gait, splayed hind limbs, and myofiber atrophy resembling PSC piglets. In addition, the absence of Pax7 results in progressive SATC loss due to apoptosis and cell cycle defects (25). Thus, in the context of early postnatal growth, Pax7 is essential for the survival of juvenile cycling muscle progenitors (25, 59). Similar to our study, apoptosis starts directly after birth in the Pax7^−/−^ model used by Relaix et al. (25). They showed that cell death occurred in active myoblasts marked by desmin. Therefore, reduced desmin expression in the AA genotype of Homer1 might at least in part reflect the increased apoptotic activity that we found in muscle samples from PCS piglets. In addition to its anti-apoptotic function, Pax7 is indispensable for the expansion of juvenile muscle progenitors, while maintaining their myogenic potential (23, 25). Specifically, Pax7 controls the activation of MyoD and, thus, myogenesis (25). In accord, there was a trend of decreased expression of MyoD in the AA variant, which also shows the lowest expression of MyoG and desmin, which are both MyoD targets. In addition to MyoG expression, exit from the cell cycle is an essential step toward terminal differentiation (73). MyoD-dependent upregulation of the cyclin-dependent kinase inhibitor p21 is an integral component of this irreversible process (67, 68, 75). Lower MyoD expression will thus lead to reduced expression of p21, which is also known to be a critical negative regulator of both proliferation and apoptosis (32, 67). Its disruption results in increased proliferation and apoptosis but reduced myotube formation (67, 76, 77). Indeed, we found low levels of MyoD and MyoG, a higher proportion of proliferative Pax7^+^/Ki67^+^ progenitor cells, and increased apoptotic activity in muscle samples from PCS piglets. These results are in accord with those found in studies with MyoD^−^ or p21-deficient mice, indicating delayed myoblast-to-myocyte transition (66, 75, 78). In fact, our *in vitro* assays with isolated cells confirmed delayed differentiation kinetics in the PCS group.

In contrast to Pax7, Myf5 expression is not negatively affected by the AA genotype and shows a trend toward upregulation. This is in accordance with data from others showing that lowering of MyoD is associated with Myf5 upregulation (79) and that reduction of Pax7 has no effect on Myf5. Most importantly, Myf5 can activate MyoG directly, whereas activation of myogenesis by Pax7 is MyoD-dependent (25, 66). Therefore, Myf5 can partly compensate for MyoD in myogenesis regulation.

## 5 Conclusion

In this study, we show for the first time that the PCS syndrome includes a changed functionality of the early postnatal myogenic progenitor population. Our findings reveal a higher apoptotic activity, a higher proportion of proliferating committed progenitors (Pax7^+^/Ki67^+^), and a reduced differentiation potential as reflected by repressed expression of MyoD and MyoG. The imbalance between proliferation/cell death and differentiation has negative effects on postnatal hypertrophic growth and contributes to PSC-related myofiber atrophy. Our results revealed that the HOMER1_rs325197091 A-variant is over-represented in the PCS group. The homozygous AA-variant is associated with a lower expression of the Homer1-subtype 205 and of the SATC marker Pax7, which is essential for the production of functional SATC and regulation of myogenesis. On the other hand, Myf5 expression is higher in this genotype. Thus, it can be concluded that the equilibrium of Homer1 proteins and, thereby, its regulatory functions are impaired in a way detrimental to the myogenic differentiation program. Thus, our results indicated a direct link between a relevant myogenic gene and PCS pathogenesis. However, the exact mode of action for Homer1-205-dependent regulation of Pax7 expression still needs to be elucidated.

## Data availability statement

The raw data supporting the conclusions of this article will be made available by the authors, without undue reservation.

## Ethics statement

Ethical review and approval was not required for the study on animal participants in accordance with the local legislation and institutional requirements.

## Author contributions

TS and MR: conceived and designed the study. TS, HR, and SM: collected, compiled, and analyzed the data. TS, SM, and MR: drafted and edited the manuscript. All authors contributed to the article and approved the submitted version.
